# Occupational Exposure to Endocrine-Disrupting Chemicals and Birth Weight and Length of Gestation: A European Meta-Analysis

**DOI:** 10.1289/EHP208

**Published:** 2016-05-06

**Authors:** Laura Birks, Maribel Casas, Ana M. Garcia, Jan Alexander, Henrique Barros, Anna Bergström, Jens Peter Bonde, Alex Burdorf, Nathalie Costet, Asta Danileviciute, Merete Eggesbø, Mariana F. Fernández, M. Carmen González-Galarzo, Regina Gražulevičienė, Wojciech Hanke, Vincent Jaddoe, Manolis Kogevinas, Inger Kull, Aitana Lertxundi, Vasiliki Melaki, Anne-Marie Nybo Andersen, Nicolás Olea, Kinga Polanska, Franca Rusconi, Loreto Santa-Marina, Ana Cristina Santos, Tanja Vrijkotte, Daniela Zugna, Mark Nieuwenhuijsen, Sylvaine Cordier, Martine Vrijheid

**Affiliations:** 1 ISGlobal, Center for Research in Environmental Epidemiology, Barcelona, Spain; 2 Consorcio de Investigación Biomédica de Epidemiología y Salud Pública, Spain; 3 Universitat Pompeu Fabra, Barcelona, Spain; 4 Department of Preventive Medicine and Public Health, University of Valencia, Valencia, Spain; 5 Center for Research in Occupational Health, Barcelona, Spain; 6 Norwegian Institute of Public Health, Oslo, Norway; 7 Department of Clinical Epidemiology, Predictive Medicine and Public Health, University of Porto Medical School, Porto, Portugal; 8 Institute of Environmental Medicine, Karolinska Institutet, Stockholm, Sweden; 9 Department of Occupational and Environmental Medicine, Copenhagen University Hospital Bispebjerg, Copenhagen, Denmark; 10 Department of Public Health, Erasmus University Medical Center, Rotterdam, the Netherlands; 11 National Institute of Health and Medical Research, InsermU1085 – Irset, University of Rennes, Rennes, France; 12 Department of Environmental Science, Vytautas Magnus University, Kaunas, Lithuania; 13 Instituto de Investigación Biosanitaria ibs.Granada, University of Granada, Granada, Spain; 14 Department of Environmental Epidemiology, Nofer Institute of Occupational Medicine, Lodz, Poland; 15 The Generation R Study Group, Department of Epidemiology, Department of Pediatrics, Erasmus University Medical Center, Rotterdam, the Netherlands; 16 Hospital Del Mar Medical Research Institute, Barcelona, Spain; 17 National School of Public Health, Athens, Greece; 18 Sachs’ Children’s Hospital, Södersjukhuset, Stockholm, Sweden; 19 Department of Clinical Science and Education, Karolinska Institutet, Stockholm, Sweden; 20 Faculty of Medicine, University of the Basque Country, Leioa, Basque Country, Spain; 21 BioDonostia Health Research Institute, San Sebastian, Basque Country, Spain; 22 Department of Social Medicine, Faculty of Medicine, University of Crete, University Hospital of Heraklion, Crete, Greece; 23 Department of Public Health, University of Copenhagen, Copenhagen, Denmark; 24 Meyer Children’s University Hospital, Florence, Italy; 25 Department of Health, Government of the Basque Country, San Sebastian, Spain; 26 Department of Public Health, Academic Medical Center, University of Amsterdam, Amsterdam, the Netherlands; 27 Cancer Epidemiology Unit, Department of Medical Sciences, University of Turin, Turin, Italy

## Abstract

**Background:**

Women of reproductive age can be exposed to endocrine-disrupting chemicals (EDCs) at work, and exposure to EDCs in pregnancy may affect fetal growth.

**Objectives:**

We assessed whether maternal occupational exposure to EDCs during pregnancy as classified by application of a job exposure matrix was associated with birth weight, term low birth weight (LBW), length of gestation, and preterm delivery.

**Methods:**

Using individual participant data from 133,957 mother–child pairs in 13 European cohorts spanning births from 1994 through 2011, we linked maternal job titles with exposure to 10 EDC groups as assessed through a job exposure matrix. For each group, we combined the two levels of exposure categories (possible and probable) and compared birth outcomes with the unexposed group (exposure unlikely). We performed meta-analyses of cohort-specific estimates.

**Results:**

Eleven percent of pregnant women were classified as exposed to EDCs at work during pregnancy, based on job title. Classification of exposure to one or more EDC group was associated with an increased risk of term LBW [odds ratio (OR) = 1.25; 95% CI: 1.04, 1.49], as were most specific EDC groups; this association was consistent across cohorts. Further, the risk increased with increasing number of EDC groups (OR = 2.11; 95% CI: 1.10, 4.06 for exposure to four or more EDC groups). There were few associations (p < 0.05) with the other outcomes; women holding job titles classified as exposed to bisphenol A or brominated flame retardants were at higher risk for longer length of gestation.

**Conclusion:**

Results from our large population-based birth cohort design indicate that employment during pregnancy in occupations classified as possibly or probably exposed to EDCs was associated with an increased risk of term LBW.

**Citation:**

Birks L, Casas M, Garcia AM, Alexander J, Barros H, Bergström A, Bonde JP, Burdorf A, Costet N, Danileviciute A, Eggesbø M, Fernández MF, González-Galarzo MC, Gražulevičienė R, Hanke W, Jaddoe V, Kogevinas M, Kull I, Lertxundi A, Melaki V, Andersen AM, Olea N, Polanska K, Rusconi F, Santa-Marina L, Santos AC, Vrijkotte T, Zugna D, Nieuwenhuijsen M, Cordier S, Vrijheid M. 2016. Occupational exposure to endocrine-disrupting chemicals and birth weight and length of gestation: a European meta-analysis. Environ Health Perspect 124:1785–1793; http://dx.doi.org/10.1289/EHP208

## Introduction

### Background

Potential endocrine-disrupting chemicals (EDCs) have been described as human-made substances that alter hormone regulation in humans or wildlife (WHO/UNEP 2012). The endocrine system regulates many essential body functions such as growth, behavior, and reproduction through the controlled release of hormones. EDCs include many synthetic and natural chemicals such as polycyclic aromatic hydrocarbons (PAHs), polychlorinated biphenyls (PCBs), pesticides, phthalates, organic solvents, phenols such as bisphenol A (BPA), alkylphenolic compounds (APCs), brominated flame retardants (BFRs), some metals, and parabens. Human exposure to EDCs has been associated with a wide range of health outcomes such as breast, prostate, and testis cancer, diabetes, obesity, and decreased fertility (De Coster and van Larebeke 2012; McLachlan et al. 2006). Although policy regarding the use of EDCs has evolved over the years, EDCs remain present in some foods and consumer products and in the workplace (De Coster and van Larebeke 2012; WHO/UNEP 2012). Individuals in the general population are exposed to small concentrations of EDCs through diet and consumer products, but some can be exposed to substantially higher concentrations of EDCs at work (WHO/UNEP 2012).

Women make up half of the workforce, and many of them are of reproductive age (European Agency for Safety and Health at Work 2016). During pregnancy, periods of fetal vulnerability occur during growth and development of organs and systems, leaving the fetus particularly sensitive to environmental factors (Grandjean et al. 2008). This is cause for concern, given that EDCs are potentially damaging during the embryonic and fetal periods because they resemble or interfere with the hormones, neurotransmitters, growth factors, and other signaling substances that normally regulate fetal development (De Coster and van Larebeke 2012). Previous studies have evaluated the impact of maternal EDC exposure in the general population on fetal growth and found exposure associated with impaired growth (Govarts et al. 2012; Lopez-Espinosa et al. 2011; Wolff et al. 2008). However, studies of maternal occupational exposure to EDCs and fetal growth outcomes are few and limited in size (< 5,000 subjects), providing insufficient sample size to evaluate infrequent occupational exposures (Snijder et al. 2011, 2012).

### Objectives

In this study we aimed to assess whether maternal occupational exposure to EDCs as classified by a job exposure matrix was associated with birth weight, term low birth weight (LBW), length of gestation, and preterm delivery in a population of 133,957 mother–child pairs from 13 population-based birth cohorts in 11 European countries.

## Methods

### Study Population

As part of the Environmental Health Risks in European Birth Cohorts (ENRIECO) and Developing a Child Cohort Research Strategy for Europe (CHICOS) projects, data held by existing European birth cohorts were inventoried, including data on birth and child health outcomes and maternal occupation (Larsen et al. 2013; Vrijheid et al. 2012). Among these birth cohorts, 13 participated in a previous study regarding maternal occupations and birth outcomes (Casas et al. 2015) and were invited to participate in this new study. All 13 birth cohorts agreed to participate, including a total of 221,837 mother–child pairs followed at birth in the cohorts from 11 different countries spanning all regions of Europe (Table 1). Informed consent was obtained from all study participants as part of the original studies and in accordance with each study’s institutional review board.

**Table 1:** Description of birth cohorts.

Cohort	Location	Time period of enrollment^*a*^	Maternal occupational history information		*n* available for analysis	*n* with history of work and ISCO88 code	*n* with unclassifiable exposure	*n* included in analysis^*b*^
			Time of collection	Period of pregnancy covered				
ABCD	The Netherlands	2003–2004	1st trimester of pregnancy	1st trimester	7,792	5,365	149	5,216
BAMSE	Sweden	1994–1996	Birth	Birth	3,883	3,536	11	3,525
DNBC	Denmark	1996–2002	12th week	1 month before conception and 1st trimester	86,736	70,015	858	69,157
Generation R	The Netherlands	2001–2006	30th pregnancy week	All trimesters until 30th week	6,444	5,207	57	5,150
Generation XXI	Portugal	2005–2006	Birth	All trimesters	7,859	5,994	338	5,656
INMA Granada	Spain	2000–2002	Birth	Birth	497	220	34	186
INMA New^*c*^	Spain	2004–2008	12th and 32nd weeks	1 month before conception and all trimesters until 32nd week	2,008	1,767	217	1,550
KANC	Lithuania	2007–2009	3rd trimester of pregnancy	1 month before conception and 1st and third trimesters	4,253	3,538	61	3,477
MoBa	Norway	1999–2008	17th pregnancy week	17th pregnancy week	93,891	31,019	827	30,192
NINFEA	Italy	2005–2011	Before maternity leave began	Variable during pregnancy	2,865	2,504	49	2,455
Pélagie	France	2002–2006	1st trimester of pregnancy	1 month before conception and 1st trimester	3,322	2,918	43	2,875
REPRO PL	Poland	2007–2011	8–12th, 20–24th, and 30–34th weeks	1 month before conception and all trimesters until 30–34th weeks	1,176	996	26	970
Rhea	Greece	2007–2008	1st and 3rd trimesters of pregnancy	1 month before conception and all trimesters	1,111	878	8	870
	Total				221,837	133,957	2,678	131,279
Birth cohorts: ABCD, Amsterdam Born Children and their Development; BAMSE, The Stockholm Children Allergy and Environmental Prospective Birth Cohort Study; DNBC, Danish National Birth Cohort; INMA, INfancia y Medio Ambiente (Childhood and Environment); KANC, Kaunas neonatal cohort; MoBa, Norwegian Mother and Child Cohort Study; NINFEA, Nascita e INFanzia: gli Effetti dell’Ambiente (Birth and Infancy: Effects of Environment); REPRO PL, Polish Mother and Child Cohort. ^***a***^All cohorts enrolled at pregnancy except for BAMSE, Generation XXI, and INMA New, which enrolled at birth. ^***b***^Mothers with exposure and outcome data. ^***c***^INMA New cohorts included the regions of Gipuzkoa, Sabadell, and Valencia.								

The population sample for the present analysis was limited to live-born infants (defined as a birth of an infant showing signs of life at a gestational age of at least 22 completed weeks or weighting ≥ 500 g), singleton pregnancies, women being employed during the period starting 1 month before conception until birth, women with occupations coded according to the International Standard Classification of Occupations of 1988 (ISCO88; http://www.ilo.org/public/english/bureau/stat/isco/isco88/), and with information on birth weight or length of gestation. From the 221,837 mother–child pairs followed at birth, 133,957 pregnant women fulfilled these criteria (Table 1). Research has shown that the active working population, particularly among women, is healthier than the nonworking population (Shah 2009) and that this is likely to result in differences in birth outcomes such as birth weight (Casas et al. 2015). Therefore, we have excluded nonworking women from our analysis.

### Occupational Exposure to EDCs

Information about whether the mother worked during the period starting 1 month before conception until birth and the corresponding job title was collected through self-reports or from questionnaires conducted by trained interviewers during pregnancy or after birth in each participating cohort (Table 1). To estimate maternal occupational exposure to EDCs during pregnancy, we linked the occupational ISCO88 codes of our population to a job exposure matrix (JEM) for EDCs (Brouwers et al. 2009). To develop this JEM, three experts expanded on the United Kingdom EDC JEM created by van Tongeren et al. (2002) and assigned exposure probability scores for all chemical groups to 353 different job titles, made for workers in the Netherlands between 2005 and 2007 (Brouwers et al. 2009). This JEM classified EDCs into 10 general chemical groups and 33 subgroups (Table 2) of those substances in which occupational exposure was expected to contribute significantly to an individual’s body burden compared to other sources such as diet. The 10 chemical groups are the following: PAHs, PCBs, pesticides, phthalates, organic solvents, BPA, APCs, BFRs, metals, and miscellaneous (benzophenones, parabens, and siloxanes); as well as a category dichotomously indicating exposure to one or more EDC groups. This JEM estimated exposure to these chemical groups for these 353 job titles with three levels of exposure probability: “unlikely,” “possible,” and “probable.” The exposure estimates refer to the occupational exposure level that would exceed the background level of exposure in the general population. This JEM makes no distinction between routes of exposure (inhalation, ingestion, or dermal). The JEM includes a fourth exposure category, “unclassifiable,” which indicates that exposure cannot be determined.

**Table 2:** Chemical groups and subgroups of substances with endocrine-disrupting potential that were used in the Brouwers et al. (2009) job exposure matrix.

Chemical group	Subgroups
Polycyclic aromatic hydrocarbons	None
Polychlorinated organic compounds	Polychlorinated biphenyls
	Dioxins, furans, polychlorinated naphthalene
	Hexachlorobenzene
	Octachlorostyrene
Pesticides	Organochlorines
	Carbamates
	Organophosphates
	Tributyltin
	Pyrethroids
	Other pesticides
Phthalates	Di(2-ethylhexyl) phthalate, di-isononyl phthalate, di-*n*-hexyl phthalate
	Benzylbutyl phthalate
	Dibutyl phthalate
	Diethyl phthalate
Organic solvents	Ethylene glycol ethers
	Styrene
	Toluene
	Xylene
	Trichloroethylene
	Perchloroethylene
Bisphenol A	None
Alkylphenolic compounds	Alkylphenolic ethoxylates
	Alkylphenols
Brominated flame retardants	Tetrabromobisphenol A
	Hexabromocyclodecane
	Polybrominated bisphenyl ethers
Metals	Arsenic
	Cadmium
	Copper
	Lead
	Mercury
Miscellaneous	Benzophenones
	Parabens
	Siloxanes

Because the JEM coded occupations using the Standard Occupational Classification 2000 (SOC2000; http://www.bls.gov/soc/2000/socguide.htm) system, the JEM was first translated from SOC2000 to ISCO88 codes using the CAMSIS tool (CAMSIS 2005). Of the 133,957 women who had occupational history available and had an ISCO88 job code, the JEM provided exposure estimates for 95,280 women and labeled 2,585 women as exposure unclassifiable (Table 3). For the remaining 36,092 women in our population, three occupational experts (S.C., A.M.G., and M.N.) evaluated their corresponding ISCO88 codes and assigned a similar ISCO88 code for which a JEM score was available. For example, our translated JEM did not provide a score for the occupation “chemical engineering technicians” (ISCO88 code 3116); therefore our occupational experts assigned a proxy ISCO88 code that was in our JEM, “chemical and physical science technicians” (ISCO88 code 3111), keeping in mind similar EDC exposure in the workplace (see Excel File Table S2). This yielded exposure estimates for 35,999 more women. Experts categorized 93 women as “exposure unclassifiable.” With the CAMSIS tool and experts’ input together, this yielded EDC exposure scores for 131,279 women (95,280 + 35,999) and labeled 2,678 women (2,585 + 93) as “exposure unclassifiable.” The 131,279 women for whom we could estimate exposure were used in our subsequent analysis (Table 3).

**Table 3:** Application of a job exposure matrix (JEM) and input of experts’ proxy codes.*^a^*

JEM score	Direct CAMSIS SOC2000 to ISCO88 translation available	Experts assigned proxy ISCO88 code	Total
0, 1, or 2	95,280	35,999	131,279
88	2,585	93	2,678
Total	97,865	36,092	133,957
Score key: 0 = exposure is unlikely to occur; 1 = exposure is possible for some workers but probability is low; 2 = exposure is likely to occur; 88 = job title provides too little information to classify exposure. ^***a***^Number of mothers with exposure and outcome data.			

### Birth Weight and Length of Gestation

Birth weight and length of gestation were collected through medical records. The last menstrual period (LMP)–based length of gestation estimate was used if it was consistent by ≤ 7 days with the ultrasound-based estimate. When these estimates were not consistent, or the LMP measurement was unavailable, the ultrasound-based estimate was preferred. If both measurements (LMP and ultrasound) were unavailable, the maternal reported length of gestation measurement was used.

The study focused on the following birth outcomes: birth weight (grams), term LBW (< 2,500 g vs. ≥ 2,500 g for births ≥ 37 weeks of gestation), length of gestation (days), and preterm delivery (< 37 weeks vs. ≥ 37 weeks).

### Covariate Data

Information on covariates used in this study was collected similarly in each cohort and included sex of the newborn (male, female), parity (0, 1, or ≥ 2), maternal age (continuous in years), maternal country of birth (European, non-European in cohorts where this was available and heterogeneous), marital status (living with the child’s father, or not), maternal education (low, medium, high, defined within cohorts; see Table S3), maternal active smoking during pregnancy (none, < 10 cigarettes/day, or ≥ 10 cigarettes/day), and maternal prepregnancy body mass index (BMI) (< 18.5, 18.5–24.9, 25–29.9, ≥ 30 kg/m^2^).

### Statistical Analysis

During the previous study all data were cleaned, variables were labeled, and categories were harmonized among all data sets in the 13 cohorts (Casas et al. 2015). All analyses were performed using Stata 12 statistical software (StataCorp, College Station, TX). For all associations, a *p*-value of ≤ 0.05 was used to define statistical significance.

Classification of maternal occupational exposure to EDCs overall was first evaluated by comparing exposure to one or more of the 10 EDC groups (“possible” and “probable” categories combined) with the unexposed group (“unlikely” exposure in all EDC groups), and second by comparing classified exposure to 1–3 EDC groups and ≥ 4 EDC groups with the unexposed group. Further, exposure classification (“possible” and “probable” combined) to each of the 10 specific EDC groups was compared with the same unexposed group (“unlikely” exposure in all EDC groups). Multivariate linear regression models were used for continuous variables (birth weight and length of gestation) and multivariate logistic regression models were used for dichotomous outcomes (term LBW and preterm delivery). For all models, we performed complete case analysis, including only subjects with available information on the exposure, outcome, and covariates. All models were adjusted for the following potential confounders: parity, maternal age, maternal country of birth (only in those cohorts where this was heterogeneous: ABCD, BAMSE, Generation R, INMA New, NINFEA, and Pélagie; see Table 1 for cohort names), maternal marital status, maternal education, maternal active smoking during pregnancy, maternal prepregnancy BMI, and sex of newborn. Models for birth weight and term LBW were additionally adjusted for gestational length in weeks. The associations between classified maternal occupational exposure to EDCs and birth outcomes were first estimated for each individual cohort, using the above-described covariate models, which differed between cohorts only with regard to the maternal country of birth variable. Then, the estimated effects were meta-analyzed, combining separate estimations from each cohort (Cochran 1954; Harris et al. 2008). Results of meta-analyses for term LBW and preterm birth are reported only for exposures with a total of at least five exposed cases among all of the cohorts (combined). To test heterogeneity among cohorts, we used Cochran’s *Q*-test and the *I^2^* statistic (Higgins et al. 2003; Thompson and Sharp 1999). If the *Q*-test was significant (*p* < 0.05) and/or *I^2^* ≥ 25%, random-effects analysis was used. We then used meta-regressions (Baker et al. 2009) to assess whether heterogeneity across cohorts was attributable to the timing during pregnancy when occupational history was collected (whole pregnancy period; 1st, 2nd, and 3rd trimesters; birth), the geographical region (southern cohorts: Generation XXI, INMA Granada, INMA New, NINFEA, Pélagie, and Rhea versus northern cohorts: ABCD, BAMSE, DNBC, Generation R, KANC, MoBa, and REPRO_PL), or the period of enrollment (before or after 2003). Further sensitivity analysis was performed to assess the robustness of our results by excluding DNBC and MoBa, the largest cohorts, from meta-analyses. Robustness was also explored by excluding elected cesareans and by stratifying associations by sex of the newborn, maternal education (primary or secondary versus university or more), and maternal active smoking during pregnancy (any or none) to evaluate the results in different strata of these variables.

### Role of the Funding Source

The sponsors of the study had no role in study design, data collection, data analysis, data interpretation, or writing of the report. The corresponding author had full access to all the data in the study and had final responsibility for the decision to submit for publication.

## Results

Among the 131,279 women in our analysis, the mean (± SD) birth weight for newborns was 3,541 ± 561 g. Babies in the Rhea cohort were the smallest with a mean birth weight of 3,156 ± 488 g, and babies in the MoBa cohort were the largest (3,604 ± 553 g) (Table 4). The mean length of gestation for all newborns in analysis was 39.8 ± 1.8 weeks. Newborns in the Rhea cohort had also the shortest gestational period with a mean gestational length of 38.5 ± 1.6 weeks, and newborns in the DNBC cohort had the largest length of gestation (40.0 ± 1.7 weeks) (Table 4). In 8 of the 13 cohorts, < 2% of newborns were term LBW, compared with 2.2–5.6% in the remaining cohorts (Generation XXI, INMA Granada, INMA New, NINFEA, and Rhea). The prevalence of preterm delivery was < 6%, except in Generation XXI, NINFEA, and Rhea (7.2, 6.8, and 12.9% preterm, respectively) (Table 4). The distribution of covariates across cohorts is shown in Table S4. Reported results are from complete case analysis.

**Table 4:** Distribution of birth outcomes by cohorts.*^a^* ^,^ *^b^*

Outcomes	ABCD	BAMSE	DNBC	Generation R	Generation XXI	INMA Granada	INMA New	KANC	MoBa	NINFEA	Pélagie	REPRO PL	Rhea	Total
Birth weight (g) (mean ± SD)	3,451 ± 562	3,557 ± 537	3,592 ± 561	3,454 ± 545	3,194 ± 480	3,298 ± 443	3,244 ± 486	3,489 ± 540	3,604 ± 553	3,214 ± 522	3,390 ± 486	3,368 ± 461	3,156 ± 488	3,541 ± 561
Missing (*n*)	24	0	369	10	97	1	10	0	14	0	1	0	14	540
Gestational length (weeks) (mean ± SD)	39.8 ± 1.8	39.9 ± 1.9	40 ± 1.7	39.9 ± 1.7	38.8 ± 1.7	39.3 ± 1.5	39.6 ± 1.7	39.3 ± 1.7	39.6 ± 1.8	39.4 ± 2.1	39.9 ± 1.6	39.5 ± 1.5	38.5 ± 1.6	39.8 ± 1.8
Missing (*n*)	0	0	0	1	28	2	4	0	0	2	0	1	196	234
Term low birth weight [*n* (%)]^*c*^	84 (1.7)	27 (0.8)	624 (0.9)	89 (1.8)	194 (3.7)	4 (2.2)	42 (2.8)	47 (1.4)	201 (0.7)	77 (3.4)	32 (1.2)	18 (1.9)	44 (5.6)	1,483 (1.2)
Preterm birth [*n* (%)]	271 (5.2)	170 (4.8)	3,036 (4.4)	238 (4.6)	407 (7.2)	8 (4.4)	68 (4.4)	190 (5.5)	1,358 (4.5)	166 (6.8)	99 (3.4)	43 (4.4)	87 (12.9)	6,141 (4.7)
Missing (*n*)	0	0	0	1	28	2	4	0	0	2	0	1	196	234
Total (*n*)	5,216	3,525	69,157	5,150	5,656	186	1,550	3,477	30,192	2,455	2,857	970	870	131,279
^***a***^Frequencies and percentages were calculated for categorical variables whereas mean and SD were calculated for continuous variables. ^***b***^Number of mothers with exposure and outcome data. ^***c***^For term LBW, preterm births (*n* = 6,141) are excluded from analysis.														

Overall, 11% of women held jobs that were classified as possibly or probably exposed to EDCs (Table 5). INMA New and Rhea were the cohorts with the highest proportion of women with job titles classified as exposed to EDCs at work (27% and 30%, respectively) (Table 5). Many pregnant women held jobs classified as exposed in INMA Granada and Pélagie cohorts, with 25% and 16% of pregnant women exposed, respectively. NINFEA and MoBa had the lowest proportion of maternal occupational exposure to EDCs, with 6% and 9% of women holding jobs classified as exposed, respectively. All other cohorts had an average exposure prevalence of around 11% (Table 5). A total of 116,358 mothers (89%) had jobs classified as unexposed to any EDCs at work, and these were used as reference group in all analyses (Table 5).

**Table 5:** Maternal occupational exposure to endocrine-disrupting chemical groups during pregnancy by cohorts as classified by application of a job exposure matrix to job titles [n (%)].*^a^*

Cohort	ABCD	BAMSE	DNBC	Generation R	Generation XXI	INMA Granada	INMA New	KANC	MoBa	NINFEA	Pélagie	REPRO PL	Rhea	Total
Total (*n*)	5,216	3,525	69,157	5,150	5,656	186	1,550	3,477	30,192	2,455	2,875	970	870	131,279
No occupational EDC exposure	4,715 (90.4)	3,116 (88.4)	61,124 (88.4)	4,573 (88.8)	4,731 (83.7)	140 (75.3)	1,126 (72.7)	3,092 (88.9)	27,579 (91.4)	2,300 (93.7)	2,402 (83.6)	851 (87.7)	609 (70.0)	116,358 (88.6)
Exposed to ≥ 1 EDC group	501 (9.6)	409 (11.6)	8,033 (11.6)	577 (11.2)	925 (16.4)	46 (24.7)	424 (27.4)	385 (11.1)	2,613 (8.7)	155 (6.3)	473 (16.5)	119 (12.3)	261 (30.0)	14,921 (11.4)
1–3 EDC groups	435 (8.3)	336 (9.5)	6,470 (9.4)	492 (9.6)	907 (16.0)	25 (13.4)	360 (23.2)	332 (9.6)	1,990 (6.6)	139 (5.7)	362 (12.6)	85 (8.8)	117 (13.5)	12,050 (9.2)
≥ 4 EDC groups	66 (1.3)	73 (2.1)	1,563 (2.3)	85 (1.7)	18 (0.3)	21 (11.3)	64 (4.1)	53 (1.5)	623 (2.1)	16 (0.7)	111 (3.9)	34 (3.5)	144 (16.6)	2,871 (2.2)
PAHs	159 (3.3)	52 (1.5)	1,074 (1.7)	291 (6.0)	43 (0.9)	9 (6.0)	70 (5.9)	125 (3.9)	404 (1.4)	25 (1.1)	41 (1.7)	15 (1.7)	39 (6.0)	2,347 (2.0)
Polychlorinated organic compounds	1 (0.0)	4 (0.1)	137 (0.2)	0	11 (0.2)	0	7 (0.6)	3 (0.1)	14 (0.1)	1 (0.0)	3 (0.1)	1 (0.1)	1 (0.2)	183 (0.2)
Pesticides	18 (0.5)	2 (0.5)	440 (1.8)	31 (1.5)	18 (0.7)	18 (11.4)	12 (1.6)	24 (0.9)	551 (2.7)	39 (1.8)	68 (4.5)	7 (1.7)	18 (16.6)	2,409 (2.0)
Phthalates	13 (1.5)	15 (2.3)	750 (2.6)	42 (1.9)	14 (0.8)	22 (13.6)	8 (5.6)	9 (1.9)	213 (2.2)	2 (0.7)	51 (4.6)	9 (3.8)	104 (19.3)	3,004 (2.5)
Organic solvents	260 (5.2)	245 (7.3)	4,581 (7.0)	197 (4.1)	486 (9.3)	26 (15.7)	303 (21.2)	151 (4.7)	1,240 (4.3)	59 (2.5)	297 (11.0)	63 (6.9)	192 (24.0)	8,100 (6.5)
BPA	0	1 (0.0)	35 (0.1)	0	0	0	10 (0.9)	3 (0.1)	0	1 (0.0)	7 (0.3)	2 (0.2)	0	59 (0.1)
APCs	187 (3.8)	148 (4.5)	3,006 (4.7)	130 (2.8)	760 (13.8)	30 (17.7)	251 (18.2)	123 (3.8)	1,047 (3.7)	29 (1.2)	271 (10.1)	43 (4.8)	187 (23.5)	6,212 (5.1)
BFRs	1 (0.0)	1 (0.0)	41 (0.1)	0	59 (1.2)	2 (1.4)	13 (1.1)	3 (0.1)	14 (0.1)	1 (0.0)	9 (0.4)	4 (0.5)	1 (0.2)	149 (0.1)
Metals	78 (1.6)	126 (3.9)	2,756 (4.3)	99 (2.1)	457 (8.8)	17 (10.8)	72 (6.0)	101 (3.2)	641 (2.3)	37 (1.6)	131 (5.2)	54 (6.0)	116 (16.0)	4,685 (3.9)
Miscellaneous chemicals	58 (1.2)	58 (1.8)	826 (1.3)	46 (1.0)	0	9 (6.0)	55 (4.7)	47 (1.5)	410 (1.5)	14 (0.6)	61 (2.5)	23 (2.6)	40 (6.2)	1,647 (1.4)
Abbreviations: APCs, alkylphenolic compounds; BFRs, brominated flame retardants; BPA, bisphenol A; EDC, endocrine-disrupting chemicals; PAHs, polycyclic aromatic hydrocarbons. ^***a***^Number of mothers with exposure and outcome data.														

**Figure 1:**
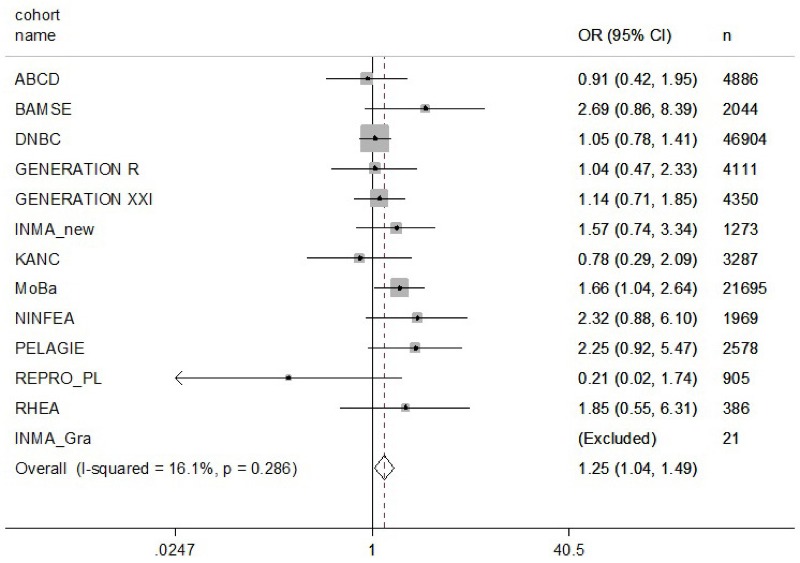
Meta-analysis of odds ratios for term low birth weight for pregnant women occupationally exposed to one or more endocrine-disrupting chemical group as classified by a job exposure matrix. N represents subjects included in complete case analysis. INMA Granada was excluded from analysis because there were no cases of term low birth weight for exposed mothers. All models are adjusted for maternal age, parity, maternal education, maternal smoking, maternal BMI, marital status, sex of newborn, and race and gestational age, where applicable. Unexposed mothers are used as reference group. Shaded boxes around the point estimates indicate the weight of the study-specific estimate.

There was no evidence for any statistically significant association with birth weight for job titles exposed to single EDC groups or for simultaneous exposure to multiple EDC groups (Table 6). The risk of delivering a term LBW baby was significantly increased among women with job titles classified as exposed to most single EDC exposure groups with odds ratios (ORs) ranging from 1.33 [95% confidence interval (CI): 1.02, 1.74] for APCs to 3.88 (95% CI: 1.37, 11.02) for BFRs (though for BFRs, this was based on only five exposed cases) (Table 6). This resulted in a 25% increased risk for delivering a term LBW baby for women holding jobs classified as exposed to one or more EDC groups (OR = 1.25; 95% CI: 1.04, 1.49) (Table 6 and Figure 1). Further, the risk increased with increasing exposure to more EDC groups at work (1–3 EDC groups: OR = 1.25; 95% CI: 1.03, 1.52; ≥ 4 EDC groups: OR = 2.11; 95% CI: 1.10, 4.06), though there was heterogeneity among cohorts for those exposed to ≥ 4 EDC groups (Table 6).

**Table 6:** Maternal occupational exposures to EDC groups during pregnancy as classified by a job exposure matrix and meta-analyzed associations (95% CI) with birth weight and length of gestation.*^a^*

Exposure	*n*^*c*^	Birth weight (g)	Term LBW^*b*^		Length of gestation (days)	Preterm delivery	
		β (95% CI)	Cases (*n*)	OR (95% CI)	β (95% CI)	Cases (*n*)	OR (95% CI)
No occupational EDC exposure	116,358	Reference	1,252	Reference	Reference	5,407	Reference
Exposed to ≥ 1 EDC group	14,921	–6.16 (–14.84, 2.51)	231	1.25 (1.04, 1.49)*	0.11 (–0.13, 0.35)	734	0.97 (0.88, 1.07)
1–3 EDC groups	12,050	–8.03 (–17.47, 1.41)	189	1.25 (1.03, 1.52)*	0.15 (–0.11, 0.42)	577	0.96 (0.86, 1.06)
≥ 4 EDC groups	2,871	0.32 (–18.68, 19.32)	42	2.11 (1.10, 4.06)^d^*	–0.05 (–0.58, 0.47)	157	1.10 (0.90, 1.35)
PAHs	2,347	–14.49 (–35.08, 6.09)	57	1.62 (1.12, 2.34)*	0.42 (–0.15, 0.99)	105	0.92 (0.73, 1.17)
PCBs	183	54.95 (–18.09, 128.00)	0	—	–0.04 (–3.51, 3.43)^*d*^	9	1.10 (0.48, 2.54)
Pesticides	2,409	1.23 (–18.98, 21.44)	33	1.85 (1.15, 2.98)*	0.01 (–1.05, 1.03)^*d*^	119	0.99 (0.78, 1.24)
Phthalates	3,004	–9.86 (–38.40, 18.69)^*d*^	45	2.35 (1.16, 4.75)^d^*	–0.02 (–0.53, 0.50)	165	1.10 (0.90, 1.34)
Organic solvents	8,100	–9.90 (–21.45, 1.66)	118	1.24 (0.97, 1.60)	0.05 (–0.27, 0.37)	420	1.05 (0.92, 1.18)
BPA	59	–66.62 (–184.16, 50.92)	3	—	3.89 (0.71, 7.07)*	1	—
APCs	6,212	–8.03 (–21.45, 5.38)	112	1.33 (1.02, 1.74)*	–0.09 (–0.62, 0.44)^*d*^	357	1.12 (0.98, 1.29)
BFRs	149	–43.48 (–117.70, 30.75)	5	3.88 (1.37, 11.02)*	2.77 (0.65, 4.89)*	6	0.92 (0.28, 3.03)
Metals	4,685	–6.39 (–20.99, 8.21)	72	1.53 (1.13, 2.07)*	0.24 (–0.17, 0.64)	236	0.96 (0.81, 1.13)
Miscellaneous	1,647	2.59 (–21.92, 27.10)	21	1.78 (0.61, 5.26)^*d*^	–0.31 (–0.99, 0.37)	88	1.17 (0.90, 1.51)
Abbreviations: —, there were < 5 exposed cases overall and meta-analysis was not completed. APCs, alkylphenolic compounds; BFRs, brominated flame retardants; BPA, bisphenol A; EDC, endocrine-disrupting chemicals; LBW, low birth weight; PAHs, polycyclic aromatic hydrocarbons; PCBs, polychlorinated organic compounds. ^***a***^For all models 116,358 unexposed mothers are used as reference group. All complete case models are adjusted for maternal age, parity, maternal education, maternal smoking, maternal BMI, marital status, sex of newborn, and race and gestational age, where applicable. ^***b***^For term LBW, preterm births (*n* = 6,141) are excluded from analysis. ^***c***^Number of mothers with exposure and outcome data. ^***d***^Heterogeneity existed among cohorts (Cochran’s *Q*-test *p* < 0.05 and/or *I*^2 ^≥ 25%), weights are from random effects analysis. *p < 0.05.							

Maternal occupations classified as exposed to BPA or BFRs during pregnancy were associated with significantly longer gestational length (3.9 days; 95% CI: 0.7, 7.1 and 2.8 days; 95% CI: 0.7, 3.0, respectively) (Table 6). Among pregnant women who held job titles with exposure to any other EDC group, no significant associations were found with gestational length or preterm delivery (Table 6).

Among significant associations, we observed heterogeneity only between occupational exposure to phthalates and term LBW; and between occupational exposure to ≥ 4 EDC groups and term LBW (Table 6; see also Figures S1 and S2). Meta-regressions revealed that this heterogeneity was not attributable to the timing during pregnancy when occupational history was collected (whole pregnancy period; 1st, 2nd, and 3rd trimesters; birth), the geographical region (southern cohorts: Generation XXI, INMA Granada, INMA New, NINFEA, Pélagie, and Rhea vs. northern cohorts: ABCD, BAMSE, DNBC, Generation R, KANC, MoBa, and REPRO_PL), or the period of enrollment (before or after 2003). Sensitivity analysis revealed that after excluding the two largest cohorts in analysis (DNBC and MoBa), associations for exposure to phthalates and ≥ 4 EDC groups and term LBW were no longer heterogeneous. Further, women with occupations classified as exposed to ≥ 4 EDC groups, PAHs, pesticides, phthalates, or metals were at an increased risk for term LBW. Exposure to BFR and risk for term LBW could not be evaluated because there were only two exposed cases. For exposures to BPA or BFR and extended length of gestation, this association lost significance for exposure to BPA and stayed the same for BFR. All other significant analyses results maintained significance and ORs of similar magnitude (see Table S5). Upon excluding women who elected cesareans (*n* = 6,889), all associations with term LBW and length of gestation were generally consistent, except for exposure to ≥ 4 EDC groups or phthalates, where ORs remained significant but weakened. Exposure to BFRs and association with term LBW lost significance (see Table S6). Stratified analyses by sex of the newborn did not change associations (Table 7). The association between exposure to one or more EDCs and term LBW was somewhat stronger in those without university education (OR = 1.32; 95% CI: 1.06, 1.64) compared to those with university education (OR = 1.24; 95% CI: 0.87, 1.77), and in smokers (OR = 1.38 95% CI: 1.01, 1.87) compared to nonsmokers (OR = 1.18; 95% CI: 0.93, 1.50) (Table 7).

**Table 7:** Stratified meta-analyses of maternal occupational exposure to one or more EDC group as classified by a job exposure matrix and odds ratios for term LBW.*^a,b^*

Stratification	Total unexposed (*n*)^*c*^	Exposed to one or more EDC group (*n*)^*c*^	Term LBW cases exposed (*n*)^*c*^	Term LBW [OR (95% CI)]
Overall	110,951	14,187	231	1.25 (1.04, 1.49)*
Sex of newborn
Male	56,590	7,240	95	1.36 (1.02, 1.81)*
Female	54,355	6,946	136	1.24 (0.97, 1.58)^*d*^
Missing	6	1	0
Maternal education
Low (primary or secondary only)	34,602	7,190	146	1.32 (1.06, 1.64)*
High (university or higher)	59,450	4,572	39	1.24 (0.87, 1.77)
Missing	16,899	2,425	46
Maternal smoking during pregnancy^*e*^
Yes	19,964	3,453	97	1.38 (1.01, 1.87)*
No	85,342	10,218	126	1.18 (0.93, 1.50)
Missing	5,645	516	8
Abbreviations: CI, confidence interval; EDC, endocrine-disrupting chemical; LBW, low birth weight; OR, odds ratio. ^***a***^For all complete case models, 110,951 unexposed mothers are used as reference group. All models are adjusted for maternal age, parity, maternal education, maternal smoking, maternal BMI, marital status, sex of newborn, and race and gestational age. ^***b***^For term LBW, preterm births (*n* = 6,141) are excluded from analysis. ^***c***^Number of subjects with exposure and outcome data. ^***d***^Heterogeneity existed among cohorts (Cochran’s *Q*-test *p* < 0.05 and/or *I*^2 ^≥ 25%). ^***e***^Yes category of smoking included any maternal smoking during pregnancy. *p < 0.05.				

## Discussion

This large meta-analysis suggests that maternal employment during pregnancy in occupations classified as possibly or probably exposed to EDCs during pregnancy is associated with a significant increased risk of term LBW in newborns, and that this association is fairly consistent across European populations and across specific groups of EDCs. We also observed that occupational exposure to BPA and BFRs as classified by the JEM was associated with significantly longer length of gestation, although few mothers were occupationally exposed (*n* = 59 and *n* = 149, respectively). Birth weight and preterm delivery were not significantly associated with JEM-classified occupational EDC exposure.

For term LBW, we found that pregnant women classified as exposed to PAHs, pesticides, phthalates, APCs, BFRs, or metals in the workplace were at significantly higher risk, and that the term LBW risk increased with increasing number of EDC groups, possibly indicating an exposure–response relationship. We restricted our analyses of LBW to term births as a way to distinguish between babies with LBW because of growth restriction and those with LBW because of early delivery. Indeed, term LBW may be a more sensitive outcome than birth weight, as suggested in relation to other environmental exposures such as air pollution (Dadvand et al. 2013; Pedersen et al. 2013).

In our study population, agricultural workers and hairdressers were classified as simultaneously exposed to at least four of these chemical groups, which made it difficult to determine whether a specific EDC group (or groups) was key for explaining associations with term LBW. It is possible also that the significant increase in risk with increasing number of EDCs is the result of other conditions related to these occupations, such as exposure to heat, unsanitary conditions, or lifting, among others (Popendorf and Donham 1991). Medical assistants, transport laborers, and waitresses were those job titles classified as exposed solely to PAHs (see Excel File Table S1). Our findings regarding occupational exposure to PAHs and term LBW agree with other studies assessing PAH exposure through air monitoring or biomarkers (Choi et al. 2006; Dejmek et al. 2000; Suzuki et al. 2010). Term LBW risk was significantly associated with pesticide exposure in our study. Agricultural workers were classified as exposed to this chemical group, among several other EDC groups, whereas pesticides was the only EDC group to which veterinarians and life science technicians were classified as exposed. In the past, exposure to pesticides among pregnant women has been widely investigated (Chevrier et al. 2011; Gemmill et al. 2013; Rauch et al. 2012; Wickerham et al. 2012), and our findings fall in line with other studies that have reported associations between reduced birth weight and maternal exposure to pesticides, both ambient and occupational (Burdorf et al. 2011; Chevrier et al. 2011; Wickerham et al. 2012; Wohlfahrt-Veje et al. 2011). However, these studies evaluated continuous birth weight, not term LBW. Agricultural workers and hairdressers were classified as being exposed to phthalates, among other chemicals, and phthalate exposure was significantly associated with term LBW. Other studies report both negative (Huang et al. 2014; Zhang et al. 2009; Zhao et al. 2015) and null (Philippat et al. 2012; Suzuki et al. 2010; Wolff et al. 2008) associations between phthalates and birth weight, but these have generally not focused on occupationally exposed populations. In our study, domestic cleaners and launderers were classified as exposed to APCs, including alklylphenols and alkylphenolic ethoxylates. Other studies regarding maternal APC exposure are rare; only the previously mentioned analysis in the Generation R cohort found a significant association with restricted fetal growth, but it did not evaluate term LBW (Snijder et al. 2012). One study in China analyzed exposure to other phenols (BPA, benzophenone-3, 4-*n*-octylphenol, and 4-*n*-nonylphenol) and found that elevated maternal levels of benzophenone-3 in urine were associated with significant reduction in gestational length only in boys, but were not significantly associated with LBW (Tang et al. 2013). More studies regarding the fetal impacts of APCs and other phenolic compounds in the general population and in the workplace are needed. The small group of mothers classified as exposed to BFRs with term LBW newborns in our study (*n* = 5) worked in plastics or textile operatives. BFRs were recently classified as EDCs by researchers at an international BFR workshop after they reviewed various publications from 2003 through 2007 (Legler 2008). Literature regarding the impact of BFRs on fetal development in humans is limited (Chen Zee et al. 2013). In our study, metals were the sole occupational EDC exposure for dental professionals, health professionals, and machine workers. Regarding exposure to metals and term LBW, our findings reflect those found in other studies regarding maternal exposure to cadmium, but in these studies, maternal exposures were not exclusively occupational (Al-Saleh et al. 2014; Sun et al. 2014; Tang et al. 2013).

Continuous birth weight was not significantly associated with any category of maternal occupational exposure to EDCs in our analysis. Previous research regarding general population exposure to EDCs and birth weight is not consistent, with varied study designs and decreases and null associations reported (Meeker 2012). Research regarding occupational exposure to EDCs during pregnancy and birth weight is very scarce. A recent study in the Generation R cohort using the same JEM found that occupational exposure to PAHs and phthalates during pregnancy was significantly associated with reduced fetal weight as estimated from ultrasounds (Snijder et al. 2012). Analyses of fetal growth measures could be a more sensitive evaluation of environmental influences during pregnancy instead of birth weight (Slama et al. 2014), but for our analysis, fetal measurements were not available for all cohorts.

Estimated exposure to BPA or BFRs was significantly associated with extended length of gestation. Workers were classified with possible or likely exposure to BPA if they held a job title as any kind of plastics operative, whereas job titles classified as exposed to BFRs were typically textile machine operators, fire service officers, or working as plastic or rubber operatives. In a smaller study (*n* = 219) embedded in the Generation R cohort, BPA in maternal urine was associated with significantly shorter gestational times or reduced fetal growth (Snijder et al. 2013), contradicting our results. However, a biomarker-based birth cohort study (*n* = 488) embedded in the INMA cohort found a small but not significant increase in length of gestation for mothers with higher levels of BPA in urine during pregnancy (Casas et al. 2016), supporting our findings. The number of pregnant women with job titles estimated as occupationally exposed to BPA or BFR (*n* = 59 and *n* = 149, respectively) among our sample was small, so these results should be interpreted with caution.

Preterm delivery was not significantly associated with estimated exposure to any EDC group in our study. Previous research regarding EDC exposure and preterm delivery is rare and has yielded conflicting results, with reports of positive, negative, and null associations with length of gestation, not necessarily preterm delivery (Meeker 2012). More research regarding this potential association, specifically among occupationally exposed mothers, is needed.

Our study has some important strengths: the harmonized and detailed information about individual maternal characteristics (e.g., parity, country of origin, marital status, education, smoking during pregnancy, and prepregnancy height and weight), enabling adjustment for potential confounders across the cohorts; the prospective data collection in most cohorts, avoiding recall bias (except BAMSE, Generation XXI, and INMA Granada; Table 1); and the large population spread throughout Europe, including data from the North, East, South and West. Previous studies of maternal occupational exposure to EDCs and associated birth weight and length of gestation have been few and relatively small and are also embedded in the Generation R study (Snijder et al. 2012, 2011). Because many cohorts participated in our study, and estimates from all participating cohorts are reported, our design also reduces the potential for publication bias, at least within the European setting. Finally, in our complete case analyses, we believe missing covariates did not influence associations. In minimally adjusted models, associations were consistent among full and complete case populations (see Table S7).

In assessing robustness of our findings, we stratified models for maternal education and maternal smoking during pregnancy, common confounders in fetal growth (Abel 1980; Kramer 1987). Associations were stronger among those with no university education and smokers (Table 7), suggesting that potential residual confounding by amount of smoking or other related factors may be present or that such factors aggravate a potential EDC effect, but this was not formally evaluated. Also, the group of exposed mothers with higher education and term LBW was relatively small (*n* = 39), so this difference may be not be meaningful. This result also may have been influenced by missing data, as education was missing for 28% of the DNBC cohort. Further, we cannot exclude residual confounding by other factors such as other maternal occupational exposures (long shifts, heavy lifting), living near sources of ambient pollution (highways, landfills), or maternal diet and physical activity during pregnancy (Brauer et al. 2008; Escribá-Agüir et al. 2001; Hegaard et al. 2007; Rodríguez-Bernal et al. 2010). We would expect these factors to act as confounders if they were also associated with the job titles grouped through the JEM. Most important, physically demanding occupations probably overlap with some of the occupations classified as exposed to EDCs, such as hairdressing, agricultural work, and waitressing. However, most of the evidence for heavy lifting relates to significant risk of preterm birth and not to term LBW (van Beukering et al. 2014). Finally, we suspect that almost all pregnant women, employed and nonemployed, are exposed to EDCs through diet and consumer products. However, this background level is believed to be much lower than occupational exposure (Nieuwenhuijsen 2003) and hence should not confound the observed associations.

Although the JEM is useful for estimating exposure for large populations when it cannot be captured via questionnaires or measurements, it is a tool meant to be used on a similar population during a similar time as that for which it was originally designed. Brouwers et al. (2009) created this particular JEM by adapting van Tongeren’s 2002 JEM (van Tongeren et al. 2002). The van Tongeren JEM was created for a UK study on workers from 1996 to 2006 (van Tongeren et al. 2002). Brouwers et al. (2009) adapted this tool to apply to a population of workers in The Netherlands between 2005 and 2007. Some of our study’s population was from the Netherlands and the majority from Northern Europe. For all cohorts in our study, most occupational data was collected between 1994 and 2013, so the windows of time for which each JEM was developed mostly align with our study population. Therefore, even though it has not been validated across countries, this JEM is the best available option for estimating occupational EDC exposure in this large sample size.

For our study, this JEM was translated from SOC2000 codes to the most relevant ISCO88 codes, and this translation was not created with EDC exposure in mind. For example, the SOC2000 job title “paramedic” was translated to the ISCO88 job title “medical assistant.” Within the JEM, paramedics were classified as exposed to PAHs because they spend much of the workday driving. This means that medical assistants in our study were classified as exposed to PAHs, which may not be accurate. With this potential for error, this could be quite magnified over a large study population resulting in broad exposure misclassification. However, we assume that such misclassification is likely to be random (nondifferential) with respect to our outcomes, and thus most likely led to attenuation of associations (Blair et al. 2007). Some studies have concluded that, in general, JEM estimates can be improved by integrating actual exposure measurements in the workplace (Teschke et al. 2002). However, it would be a large effort to measure occupational exposure to EDCs in many occupations and many European countries. We must also admit the possibility that not all women worked during the same period of pregnancy, so duration of exposure and trimesters of exposure likely differed among pregnant women. Further, because translation of Brouwers’ JEM from SOC2000 to ISCO88 codes was directly applicable only to some ISCO88 codes, we had to consult experts to estimate exposure for almost one-third (*n* = 35,999) of the women in our data set.

## Conclusions

This large-scale prospective study suggests that maternal employment during pregnancy in occupations classified as possibly or probably exposed to EDCs was associated with a significant increased risk of term LBW newborns in cohorts throughout Europe. This finding should be followed up by studying health outcomes throughout childhood and by focusing more specifically on occupations classified as exposed to multiple EDCs.

## Supplemental Material

(229 KB) PDF

(43 KB) ZIP
