# Supplementary material for: Occupational Exposure to Endocrine-Disrupting Chemicals and Birth Weight and Length of Gestation: A European Meta-Analysis
Source: Environ Health Perspect. 2016 May 6;124(11):1785–93. doi: 10.1289/EHP208 (PMC5089886; doi:10.1289/EHP208)
Supplement: (229 KB) PDF [file EHP208.s001.acco.pdf]

Environ Health Perspect
DOI: 10.1289/EHP208

Note to readers with disabilities: *EHP* strives to ensure that all journal content is accessible to all readers. However, some figures and Supplemental Material published in *EHP* articles may not conform to 508 standards due to the complexity of the information being presented. If you need assistance accessing journal content, please contact ehp508@niehs.nih.gov. Our staff will work with you to assess and meet your accessibility needs within 3 working days.

Supplemental Material

Occupational Exposure to Endocrine-Disrupting Chemicals and Birth Weight and Length of Gestation: A European Meta-Analysis

Laura Birks, Maribel Casas, Ana M. Garcia, Jan Alexander, Henrique Barros, Anna Bergström, Jens Peter Bonde, Alex Burdorf, Nathalie Costet, Asta Danileviciute, Merete Eggesbø, Mariana F. Fernández, M. Carmen González-Galarzo, Regina Gražulevičienė, Wojciech Hanke, Vincent Jaddoe, Manolis Kogevinas, Inger Kull, Aitana Lertxundi, Vasiliki Melaki, Anne-Marie Nybo Andersen, Nicolás Olea, Kinga Polanska, Franca Rusconi, Loreto Santa-Marina, Ana Cristina Santos, Tanja Vrijkotte, Daniela Zugna, Mark Nieuwenhuijsen, Sylvaine Cordier, and Martine Vrijheid

Table of Contents

Excel File Table S1: See "Additional Files" below.
Excel File Table S2: See "Additional Files" below.
Table S3: Coding of maternal education level by cohorts
Table S4: Distribution of covariates by cohorts
Table S5: Maternal occupational exposures to EDC groups during pregnancy as classified by a job exposure matrix and meta-analyzed associations (95%CI) with birth term LBW and length of gestation[a], excluding DNBC and MoBa cohorts
Table S6: Maternal occupational exposures to EDC groups during pregnancy as classified by a job exposure matrix and meta-analyzed associations 95%CI) with birth term LBW and length of gestation[a], excluding elected cesarean births
Table S7: Minimally adjusted models for maternal exposure to endocrine disrupting chemicals as classified by a job exposure matrix and associations with
Figure S1: Meta-analysis of odds ratios for term low birth weight for pregnant women occupationally exposed to 4 or more endocrine-disrupting chemical groups as classified by a job exposure matrix. N's

represent subjects included in complete case analysis. Cohorts excluded had no cases of term LBW among pregnant women classified as occupationally exposed to 4 or more endocrine-disrupting chemical groups. All models are adjusted for maternal age, parity, maternal education, maternal smoking, maternal BMI, marital status, sex of newborn, and race and gestational age, where applicable. Unexposed mothers are used as reference group. Shaded boxes around the point estimates indicate the weight of the study-specific estimate.

Figure S2: Meta-analysis of odds ratios for term LBW for pregnant women occupationally exposed to phthalates as classified by a job exposure matrix. N's represent subjects included in complete case analysis. Cohorts excluded had no cases of term LBW among pregnant women classified as occupationally exposed to 4 or more endocrine-disrupting chemical groups, except in Generation XXI, which had one case. All models are adjusted for maternal age, parity, maternal education, maternal smoking, maternal BMI, marital status, sex of newborn, and race and gestational age, where applicable. Unexposed mothers are used as reference group. Shaded boxes around the point estimates indicate the weight of the study-specific estimate.

Additional Files

- Supplemental Code and Data Zip File
  - Supplemental Code and Data Zip File Index
  - **Excel File Table S1:** Application of Brouwers 2009 JEM to ISCO88 and SOC2000 codes using CAMSIS translation with exposure score
  - **Excel File Table S2:** Experts' assignment of proxy codes for use with the Brouwers 2009 JEM

Excel File Table S1: Application of Brouwers 2009 JEM to ISCO88 and SOC2000 codes using CAMSIS translation with exposure score (see Supplemental Code and Data Zip File for this table)

Excel File Table S2: Experts' assignment of proxy codes for use with the Brouwers 2009 JEM (See Supplemental Code and Data Zip File for this table).

Table S3: Coding of maternal education level by cohorts

Cohort	Low education	Medium Education	High education
ABCD	0-5 years after primary education	6-10 years	≥11 years
BAMSE	9-year primary school/2 year secondary school	3-4 years secondary school	University or college degree
DNBC	Primary school	Lower secondary school	Upper secondary school
Generation R	No education, primary school, lower vocational training, intermediate general school or 3 years or less of general secondary school	More than 3 years of general secondary school, intermediate vocational training, or first year of higher vocational training and bachelor's degree	University degree
Generation XXI	≥6 to <9 years	≥9 to <12 years	≥12 years
INMA Granada	No education or primary school	Secondary school	University degree or higher
INMA New	No education or primary school	Secondary school	University degree or higher
KANC	No education or primary school (<12 years)	12 years- Secondary school	>12 years - University degree or higher
MoBa	<12 years	12 years- Secondary school	>12 years - University degree or higher
NINFEA	No education or primary school (≤8 years)	Secondary school (9 to 13 years)	University or higher (>13 years)
PELAGIE	<12 years	12-14 years	>14 years
REPRO PL	≤9 years	10-12 years	>12 years
RHEA	Compulsory education (up to 9 years)	Lyceum and/or Post-secondary (3-5 additional years)	University degree or higher (≥16 years)

Table S4: Distribution of covariates by cohorts[a]

Covariates	ABCD	BAMSE	DNBC[b]	Generation R	Generation XXI	INMA Granada	INMA New[c]	KANC	MoBa	NINFEA	PELAGIE	REPRO PL	RHEA	Total
Maternal age (years) mean (SD)	31.7 (4.5)	30.8 (4.4)	30.2 (4.2)	30.8 (4.7)	29.5 (5.1)	31.0 (4.7)	31.7 (4.1)	28.7 (4.8)	30.2 (4.4)	33.6 (4.0)	30.1 (4.2)	29.0 (4.1)	30.0 (4.8)	30.3 (4.4)
missing (n)	0	0	50	0	2	0	64	0	0	0	0	2	3	121
Maternal overweight or obese (%)	1,022 (19.6)	642 (20.9)	18,519 (27.2)	1,090 (24.7)	1,642 (31.6)	41 (22.16)	386 (24.9)	1,447 (41.6)	8,769 (29.8)	441 (18.4)	484 (16.9)	161 (16.7)	276 (31.8)	34,920 (27.3)
missing (n)	0	458	1,059	735	452	1	0	0	713	55	18	8	2	3,501
Maternal university education or higher (%)	2,549 (49.1)	1,491 (42.5)	33,752 (67.7)	1,543 (30.8)	1,432 (27.1)	41 (22.2)	555 (35.9)	1,969 (56.6)	20,047 (67.6)	1,494 (60.9)	1,105 (38.5)	598 (61.7)	289 (33.3)	66,865 (60.3)
missing (n)	20	13	19,274	137	362	1	3	0	531	3	3	0	2	20,349
Any maternal smoking during pregnancy (%)	467 (9.0)	431 (12.2)	16,834 (24.4)	1,110 (23.9)	1,200 (21.5)	55 (29.9)	504 (33.1)	204 (5.9)	2,582 (10.6)	184 (7.6)	778 (27.1)	13 6 (14.0)	195 (22.5)	24,680 (19.8)
missing (n)	2	1	26	499	82	2	29	0	5,823	23	2	0	4	6,493
Marital status (% living with father)	4,641 (89.1)	3,098 (96.3)	38,443 (55.6[b])	4,476 (90.4)	5,393 (95.7)	160 (98.8)	1,525 (98.5)	2,875 (82.7)	29,545 (97.9)	2,198 (98.9)	2,820 (98.3)	781 (80.5)	853 (98.3)	96,808 (74.2)
missing (n)	8	307	50	196	20	24	1	0	13	233	5	0	2	859
Gender of newborn (% male)	2,609 (50.0)	1,801 (51.1)	35,454 (51.3)	2,593 (50.4)	2,903 (51.3)	186 (100.0[c])	770 (49.8)	1,791 (51.5)	15,448 (51.2)	1,218 (49.6)	1,451 (50.5)	486 (50.2)	460 (52.9)	67,167 (51.2)
missing (n)	1	0	0	0	0	0	5	0	0	0	1	2	0	9
Parity (% first child)	3,157 (60.5)	2,055 (59.4)	32,887 (47.6)	3,199 (62.4)	3,303 (59.2)	52 (28.4)	901 (58.2)	48.8	48.2	76.8	47.0	59.3	43.5	50.3
missing (n)	0	64	45	19	77	3	2	0	0	11	5	0	23	249
Total (n)	5,216	3,525	69,157	5,150	5,656	186	1,550	3,477	30,192	2,455	2,857	970	870	131,279

Abbreviations: SD: standard deviation.
[a]Frequencies and percentages were calculated for categorical variables whereas mean and SD were calculated for continuous variables.
[b]DNBC classified mothers married or unmarried, instead of living with father or not.
[c]Enrollment in INMA Granada cohort was limited to mothers of newborn males.

Table S5: Maternal occupational exposures to EDC groups during pregnancy as classified by a job exposure matrix and meta-analyzed associations (95%CI) with birth term LBW and length of gestation[a], excluding DNBC and MoBa cohorts

Exposure	Term LBW[b]			Length of gestation (days)
	n	exposed cases	OR (95% CI)	β (95% CI)
≥ 1 EDC group	4,275	115	1.30 (1.00, 1.69)*	0.17 (-0.26, 0.60)
1 to 3 EDC groups	3,590	97	1.23 (0.93, 1.62)	0.28 (-0.18, 0.74)
4 or more groups	685	18	3.66 (1.91, 7.03)*	-0.31 (-1.28, 0.67)
PAHs	869	30	1.92 (1.19, 3.11)*	0.22 (-0.64, 1.08)
PCBs	32	0	-	0.90 (-5.25, 7.05)[c]
Pesticides	500	10	3.44 (1.32, 8.94)*	-0.11 (-1.84, 1.61)[c]
Phthalates	731	20	4.22 (2.19, 8.15)*	-0.28 (-1.28, 0.72)
Organic solvents	2,279	52	1.21 (0.81, 1.79)	0.14 (-0.43, 0.71)
BPA	24	2	-	3.46 (-0.83, 7.75)
APCs	2,159	66	1.43 (0.99, 2.05)	-0.15 (-0.98, 0.68)
BFRs	94	3	-	2.60 (0.07, 5.13)*
Metals	1,288	42	1.85 (1.22, 2.81)*	0.23 (-0.49, 0.96)
Miscellaneous	411	10	4.28 (1.85, 9.91)*	-0.47 (-1.97, 1.03)

Abbreviations: APCs: alkylphenolic compounds; BFRs: brominated flame retardants; BPA: bisphenol A; EDC: Endocrine disrupting chemicals; LBW: low birth weight; PAHs: Polycyclic aromatic hydrocarbons; PCBs, polychlorinated organic compounds.
*p<0.05
[a]For all complete case models 27,655 unexposed mothers are used as reference group. All complete case models are adjusted for maternal age, parity, maternal education, maternal smoking, maternal BMI, marital status, sex of newborn, and race and gestational age, where applicable.
[b]For term LBW, preterm births (n=1,747) are excluded from analysis.
[c]Heterogeneity existed among cohorts (Cochran's Q test p<0.05 and/or I^2≥25%), weights are from random effects analysis.
-Blank cells indicate there were less than 5 exposed cases overall and meta-analysis was not completed

Table S6: Maternal occupational exposures to EDC groups during pregnancy as classified by a job exposure matrix and meta-analyzed associations 95%CI) with birth term LBW and length of gestation[a], excluding elected cesarean births

EDC group		Term LBW[b]		Length of gestation (days)
		exposed cases	OR (95% CI)	β (95% CI)
≥ 1 EDC group	14,164	210	1.23 (1.02, 1.49)*	0.07 (-0.17, 0.31)
1 to 3 EDC groups	11,448	174	1.25 (1.02, 1.53)*	0.12 (-0.15, 0.39)
4 or more groups	2,716	37	1.94 (1.02, 3.71)[c]*	-0.12 (-0.66, 0.41)
PAHs	2,219	51	1.69 (1.14, 2.49)*	0.45 (-0.13, 1.02)
PCBs	162	0	-	-0.69 (-2.71, 1.32)
Pesticides	2,313	31	1.74 (1.06, 2.87)*	-0.06 (-1.15, 1.02)[c]
Phthalates	2,841	40	1.71 (1.08, 2.71)*	-0.09 (-0.61, 0.43)
Organic solvents	7,665	107	1.22 (0.94, 1.59)	0.03 (-0.29, 0.36)
BPA	54	3	-	3.67 (0.37, 6.97)*
APCs	5,872	105	1.32 (1.00, 1.75)*	-0.16 (-0.54, 0.23)
BFRs	137	5	2.47 (0.40, 15.25)[c]	2.81 (0.61, 5.00)*
Metals	4,454	70	1.54 (1.13, 2.10)*	0.25 (-0.42, 0.93)[c]
Miscellaneous	1,552	18	1.77 (0.59, 5.31)[c]	-0.32 (-1.01, 0.37)

Abbreviations: APCs: alkylphenolic compounds; BFRs: brominated flame retardants; BPA: bisphenol A; EDC: Endocrine disrupting chemicals; LBW: low birth weight; PAHs: Polycyclic aromatic hydrocarbons; PCBs, polychlorinated organic compounds.

*$p<0.05$

[a]For all complete case models 110,226 unexposed mothers are used as reference group. All complete case models are adjusted for maternal age, parity, maternal education, maternal smoking, maternal BMI, marital status, sex of newborn, and race and gestational age, where applicable.

[b]For term LBW, preterm births (n=5,626) are excluded from analysis.

[c]Heterogeneity existed among cohorts (Cochran's Q test $p<0.05$ and/or $I^2 \geq 25\%$), weights are from random effects analysis.

-Blank cells indicate there were less than 5 exposed cases overall and meta-analysis was not completed

Table S7: Minimally adjusted models for maternal exposure to endocrine disrupting chemicals as classified by a job exposure matrix and associations with birth weight and length of gestation.[a]

Exposure			Birth weight (g)		Term LBW[b]				Length of gestation (days)		Preterm delivery			
	n full population	n complete case	β (95% CI) full population	β (95% CI) complete case population	cases	OR (95% CI) full population	cases	OR (95% CI) complete case population	β (95% CI) full population	β (95% CI) complete case population	cases	OR (95% CI) full population	cases	OR (95% CI) complete case population
No occupational EDC exposure	116,358	88,644			1,252		955				5,407		4,018	
Exposed to ≥ 1 EDC group	14,921	11,207	-18.23 (-25.91, -10.56)*	-16.45 (-25.18, -7.73)*	231	1.42 (1.22, 1.65)*	167	1.40 (1.17, 1.66)*	-0.14 (-0.35, 0.07)	-0.05 (-0.29, 0.18)	734	1.04 (0.96, 1.13)	529	1.03 (0.94, 1.14)
1-3 EDC groups	12,050	9,099	-21.00 (-29.43, -12.57)*	-17.79 (-27.36, -8.22)*	189	1.41 (1.20, 1.66)*	140	1.41 (1.17, 1.70)*	-0.08 (-0.31, 0.16)	0.02 (-0.24, 0.28)	577	1.02 (0.93, 1.11)	414	1.01 (0.91, 1.12)
4 or + EDC groups	2,871	2,108	-7.52 (-24.30, 9.26)[c]	-11.93 (-31.15, 7.28)[c]	42	2.04 (1.30, 3.20)[c]*	27	2.12 (1.16, 3.87)[c]*	-0.38 (-0.84, 0.08)	-0.35 (-0.86, 0.17)	157	1.20 (1.01, 1.41)*	115	1.22 (1.00, 1.48)*
PAHs	2,347	1,765	-57.97 (-76.34, -39.60)[c]*	-50.94 (-75.08, -26.79)[c] *	57	2.37 (1.78, 3.16)*	39	2.14 (1.51, 3.03)*	-0.03 (-0.54, 0.47)	0.00 (-0.56, 0.56)	105	0.99 (0.81, 1.21)	83	1.08 (0.86, 1.36)
PCBs	183	136	34.34 (-31.32, 99.99)	37.09 (-38.15, 112.34)	0	-	0	-	-0.68 (-2.48, 1.13)	-0.87 (-2.85, 1.11)	9	1.10 (0.54, 2.25)	7	-
Pesticides	2,409	1,811	12.40 (-5.87, 30.67)	7.15 (-13.53, 27.84)	33	1.86 (1.27, 2.71)*	22	1.74 (1.10, 2.74)*	-0.31 (-1.18, 0.56)[c]	-0.22 (-1.26, 0.81)[c]	119	1.09 (0.90, 1.32)	85	1.04 (0.83, 1.31)
Phthalates	3,004	2,209	-8.49 (-24.87, 7.88)[c]	-11.98 (-30.73, 6.77)[c]	45	2.09 (1.30, 3.37)[c]*	29	2.21 (1.16, 4.23)[c]*	-0.34 (-0.79, 0.11)	-0.30 (-0.80, 0.21)	165	1.19 (1.01, 1.40)*	120	1.20 (0.99, 1.45)
Organic solvents	8,100	6,020	-17.48 (-27.62, -7.34)*	-16.07 (-27.65, -4.49)*	118	1.38 (1.12, 1.68)*	81	1.35 (1.06, 1.72)*	-0.19 (-0.48, 0.09)	-0.14 (-0.49, 0.22)	420	1.10 (1.00, 1.22)*	304	1.11 (0.98, 1.25)
BPA	59	48	-83.00 (-194.47, 28.47)	-78.76 (-198.68, 41.16)	3	-	3	-	2.68 (-0.40, 5.76)	3.70 (0.53, 6.86)*	1	-	0	-
APCs	6,212	4,497	-18.43 (-29.86, -7.00)*	-15.80 (-28.98, -2.62)*	112	1.54 (1.25, 1.90)*	77	1.55 (1.21, 1.99)*	-0.33 (-0.82, 0.15)	-0.40 (-1.00, 0.20)	357	1.19 (1.06, 1.33)*	255	1.21 (1.06, 1.38)*
BFRs	149	115	-39.73 (-107.46, 28.00)	-52.68 (-128.08, 22.73)	5	4.21 (1.60, 11.03)*	5	5.49 (2.07, 14.52)*	2.10 (0.16, 4.04)*	2.74 (0.64, 4.84)*	6	0.92 (0.40, 2.10)	3	-
Metals	4,685	3,521	-3.73 (-16.84, 9.38)	-6.17 (-21.05, 8.71)	72	1.47 (1.14, 1.91)*	57	1.62 (1.22, 2.16)*	-0.05 (-0.42, 0.31)	0.10 (-0.58, 0.77)	236	1.06 (0.92, 1.21)	162	1.00 (0.85, 1.18)
Miscellaneous	1,647	1,222	-23.70 (-45.51, -1.89)*	-31.84 (-62.28, -1.39)*	21	2.23 (0.94, 5.29)[c]	14	2.10 (0.74, 5.98)[c]	-0.41 (-1.33, 0.51)[c]	-0.55 (1.54, 0.45)[c]	88	1.22 (0.98, 1.52)	65	1.30 (1.01, 1.67)*

Abbreviations: APCs: alkylphenolic compounds; BFRs: brominated flame retardants; BPA: bisphenol A; EDC: endocrine disrupting chemicals; LBW: low birth weight; PAHs: polycyclic aromatic hydrocarbons; PCBs, polychlorinated organic compounds.

**p*<0.05

[a]For all models 116,358 unexposed mothers are used as reference group. All models are minimally adjusted for sex of newborn and gestational age, where applicable.

[b]For term LBW, preterm births (n=6,889) are excluded from analysis.

[c]Heterogeneity existed among cohorts (Cochran's *Q* test p<0.05 and/or I^2≥25%), weights are from random effects analysis.

-Blank cells indicate there were less than 5 exposed cases overall and meta-analysis was not completed

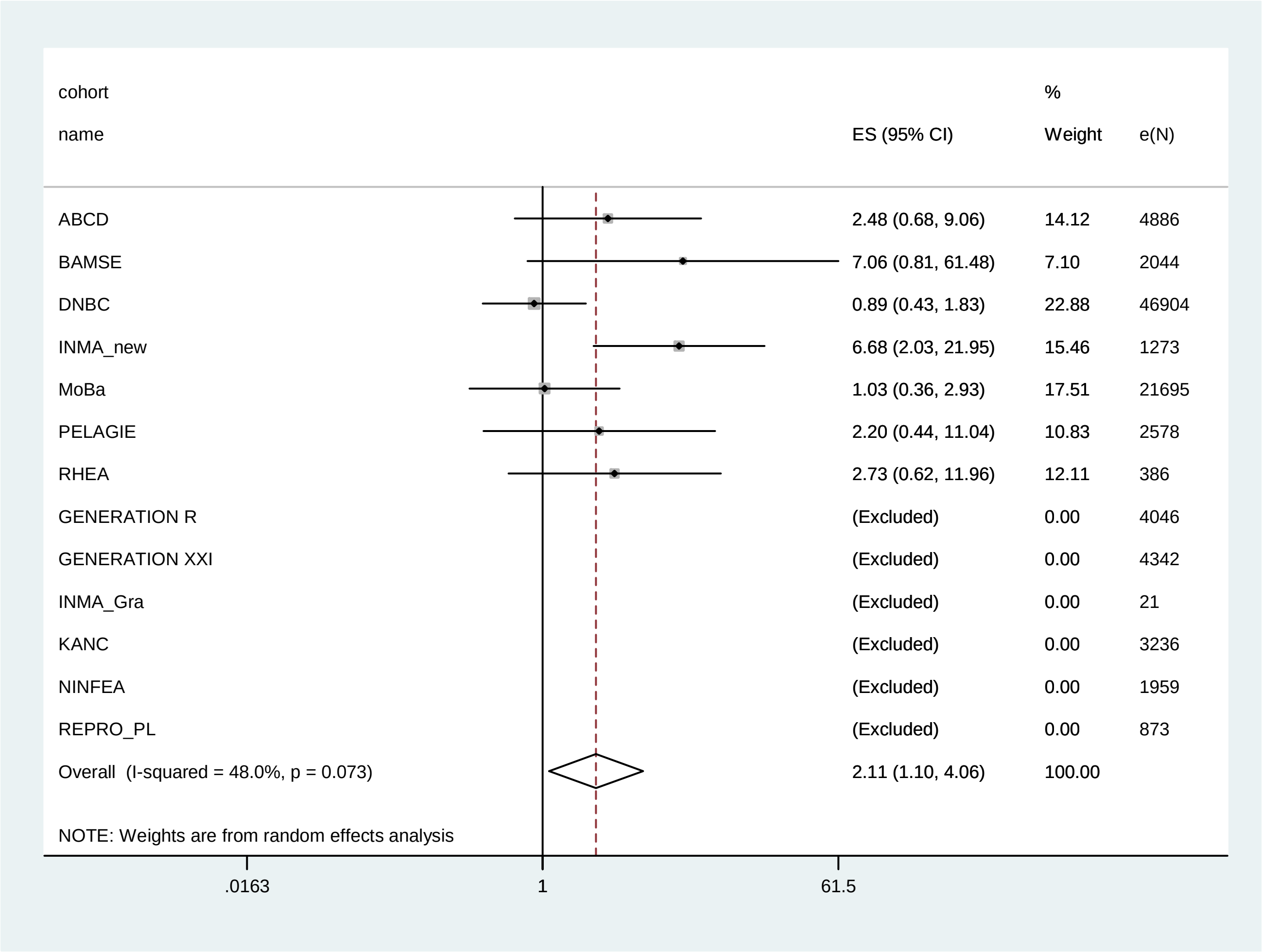

Figure S1: Meta-analysis of odds ratios for term low birth weight for pregnant women occupationally exposed to 4 or more endocrine-disrupting chemical groups as classified by a job exposure matrix. N's represent subjects included in complete case analysis. Cohorts excluded had no cases of term LBW among pregnant women classified as occupationally exposed to 4 or more endocrine-disrupting chemical groups. All models are adjusted for maternal age, parity, maternal education, maternal smoking, maternal BMI, marital status, sex of newborn, and race and gestational age, where applicable. Unexposed mothers are used as reference group. Shaded boxes around the point estimates indicate the weight of the study-specific estimate.

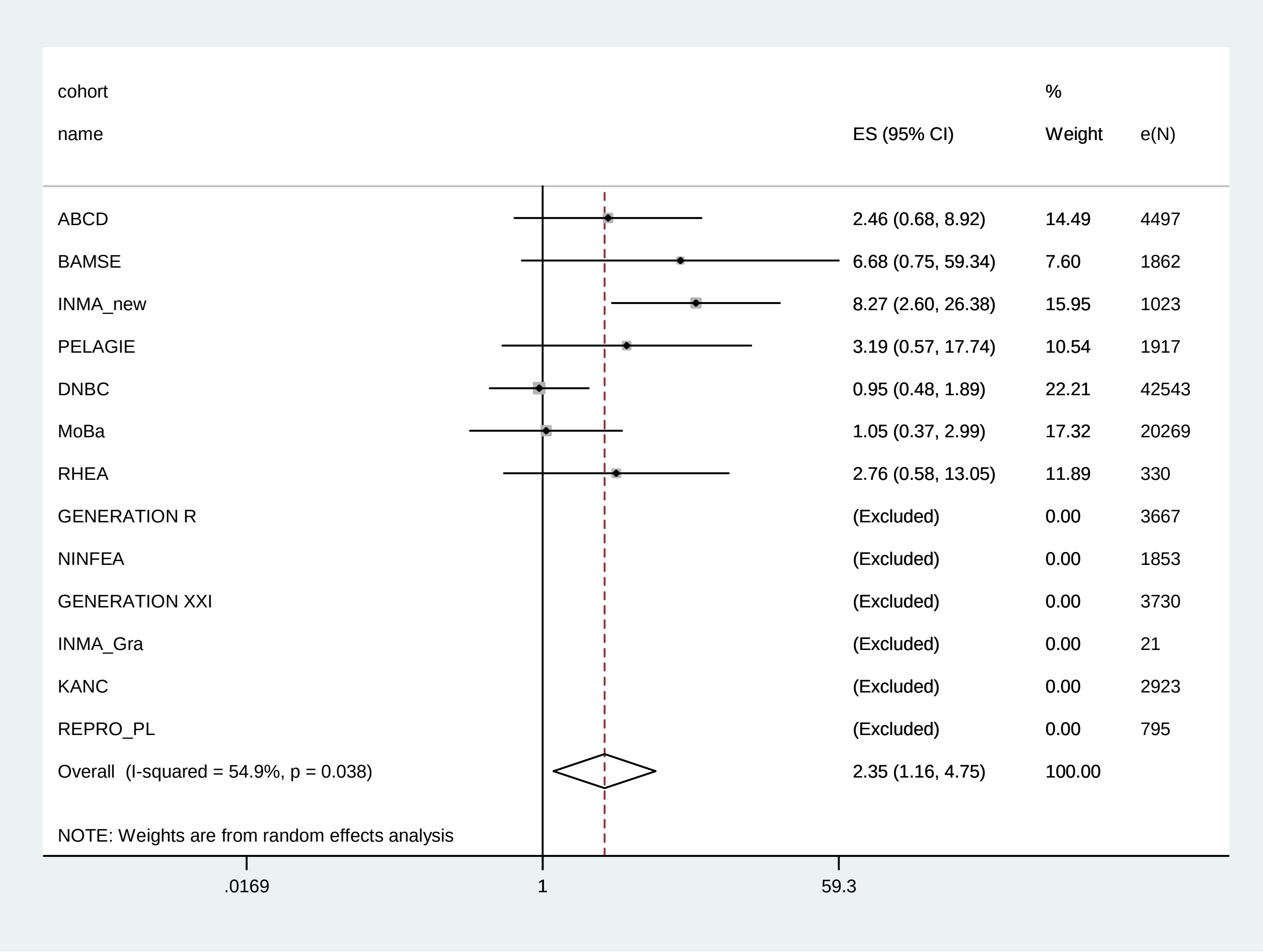

Figure S2: Meta-analysis of odds ratios for term LBW for pregnant women occupationally exposed to phthalates as classified by a job exposure matrix. N's represent subjects included in complete case analysis. Cohorts excluded had no cases of term LBW among pregnant women classified as occupationally exposed to 4 or more endocrine-disrupting chemical groups, except in Generation XXI, which had one case. All models are adjusted for maternal age, parity, maternal education, maternal smoking, maternal BMI, marital status, sex of newborn, and race and gestational age, where applicable. Unexposed mothers are used as reference group. Shaded boxes around the point estimates indicate the weight of the study-specific estimate.
